# Genetic Research and Plant Breeding 2.0

**DOI:** 10.3390/genes15121604

**Published:** 2024-12-15

**Authors:** Kwon-Kyoo Kang, Yu-Jin Jung, Yong-Gu Cho

**Affiliations:** 1 Division of Horticultural Biotechnology, Hankyong National University, Anseong 17579, Republic of Korea; yuyu1216@hknu.ac.kr; 2 Department of Crop Science, Chungbuk National University, Cheongju 28644, Republic of Korea; ygcho@cbnu.ac.kr

Recent advances in next-generation sequencing technologies have significantly reduced sequencing costs, resulting in the creation of large-scale genomic data that can be utilized for plant breeding. These technological advances have enabled precise breeding based on dielectric data, which has revolutionized existing phenotype-based selection methods [1]. Molecular breeding enables the rapid development of climate change-resistant crop varieties by leveraging genetic information. Drought, floods, and extreme temperature fluctuations caused by climate change significantly impact crop production, and there is an urgent need to develop varieties that can respond to these environmental stressors [2]. This can be achieved through molecular breeding to improve the stability and sustainability of agriculture. This enables improved agricultural productivity and the efficient use of resources, enabling high yields while minimizing environmental impact. Furthermore, while traditional breeding methods are time-consuming and costly, molecular breeding can utilize molecular markers and NGS techniques to significantly shorten the breeding process. These techniques reduce the time required to develop new varieties and respond quickly to fast-changing environments [3]. The recent introduction of artificial intelligence (AI) technology in plant breeding has further highlighted the need for molecular breeding. AI technology plays an important role in optimizing agricultural activities by exploiting data routes obtained from vast amounts of agricultural information [4]. For example, based on AI technology, the growth status of crops, the nutritional status of soil, weather conditions, etc., are monitored and predicted in real time to analyze crop growth patterns and derive optimal cultivation conditions. This allows us to predict the likelihood of pest outbreaks and enables pre-emptive responses to maximize agricultural efficiency. Therefore, integrated analyses of genetic and environmental data obtained from molecular breeding can be used to predict the genetic characteristics and environmental adaptability of crops and to design optimal mating strategies [5]. Utilizing these technologies can help shorten breeding times, reduce costs, and contribute to the development of more stable crop varieties. The molecular blending of crops requires studies of protein function and interactions between proteins using vast amounts of genetic data obtained from plant genomes. This requires a variety of advanced tools and methods, such as transcriptomic analysis, metabolomics, overexpression, knockout, RNAi, and advanced computational methodologies such as machine learning and deep learning, to effectively and efficiently validate gene functionality when working in laboratory environments [6,7]. Indeed, the *Genes* journal contains vast amounts of information for the development of new varieties based on molecular breeding. In particular, the six contributions included in this Special Issue, including one review paper, discussing the genetic analysis of yield growth, quality improvement, and stress resistance.

Simões et al. [8] published a review paper on the high-added-value genetics of stone pine (*Pinus pinea* L.). Genomic technology has the potential to contribute to deciphering the stone pine genome and developing a more resilient bioeconomy. Retrotransposons and specific genetic markers are effective tools in determining population-specific genomic diversity. Studies of transcripts and proteins have identified the differentially expressed genes *PAS1*, *CLV1*, *ATAF1*, and *ACBF* involved in bud formation. Stone pine proteomes exhibit variations between populations and demonstrate the industrial potential of pinosylvin enzymes. Microsatellite studies have shown that stone pine’s low level of polymorphism and unique genetic diversity can contribute to environmental adaptation. Transcriptomic and protein body analyses reveal stone pine’s genetic and molecular responses to fungal and nematode infections and elucidate its potential role in defense activation, gene regulation, and pathogen resistance. Transcriptomics associated with carbohydrate metabolism, dehydrogenation, and transcription factors demonstrate the potential to improve stone pine’s drought stress response and ability to retain moisture. Due to these characteristics, the stone pine has established itself as an important model tree for studying climate change adaptation. While knowledge gaps exist, stone pine’s genetic resources have significant potential, and ongoing advances in technology offer prospects for their future exploration. Additionally, Yoon et al. [9] performed QTL mapping of tiller numbers in Korean Japonica rice varieties. Quantitative trait loci (QTLs) were identified by analyzing 160 recombinant inbreeding lines (RILs) derived from a cross of Odae and Unbong 40, a temperate Japonica variety, under greenhouse conditions. QTN3, the major QTL for tiller number, was identified as 132.4 cm on chromosome 3. This QTL was also detected under field conditions in the backcross population, resulting in stable production and a stable environment. It was found that QTN3 is located with QTLs associated with the number of panicles per plant and the culm strength, which has a multilateral expression effect. Therefore, the QTL QTN3 is thought to be useful in breeding programs that develop japonica varieties with an optimal number of tillers for increased yield. Li et al. [10] analyzed the association of tiller-related traits with EST-SSR markers in *Psathyrostachys juncea*. Using 127 pairs of EST-SSR markers, the researchers analyzed the genetic diversity, population structure, and linkage disequilibrium of 480 individual lines, and found a significant association between 10 plant architecture-related traits of *P. juncea* and molecular markers. A genetic diversity analysis revealed an observational allele number of 1.957, an effective allele number of 1.682, Shannon’s information index of 0.554, observational heterozygosity of 0.353, expected heterozygosity of 0.379, and polymorphic information content of 0.300. A total of 480 individual lineages were clustered into five groups based on the nonoverlapping pair group method via population genetic structure, coordination analysis, and arithmetic mean analysis (UPGMA). The linkage disequilibrium coefficient (r^2^) ranged from 0.00 to 0.68, indicating a relatively low level of linkage disequilibrium between loci. An association analysis revealed 55 significant marker-characteristic associations (MTAs). In addition, nine SSR markers were associated with multiple traits. These results suggest that the proposed tool has promising applications in the molecular selection and breeding of *P. juncea* germplasms. 

Cucumber powder fungus (CPM) caused by *Sphaerotheca fusc* is a serious airborne disease that affects cucumbers. This disease is very difficult to manage, as conidia can attach to the host plant and induce germination. Kim et al. [11] enhanced the activation of defense-related genes and salicylic acid in cucumbers through beta-aminobutyric acid and powdered fungal infections (*Cumis sativus* L.). Studies were conducted to find defense genes induced by beta-aminobutyric acid (BABA) and powdered fungi in cucumbers. In a quantitative real-time PCR assay, the transcriptional levels of the defense genes *CsPAL*, *CsPR3*, *CsPR1*, *CsPR1*, *CsLOX1*, *CsLOX23*, *CsWRK20*, and *Cupi4* were increased to maximum levels from 48 h, *CsLecRK6.1* reached the maximum expression levels after 24 h, and salicylic acid (SA) levels were significantly increased in BABA-treated cucumber plants. In addition, *Sphagnum fuscum*-infected cucumbers showed 1.6- to 47.3-fold improvement in the defense genes *PAL*, *PR3*, *PR1*, *Lox1*, *Lox23*, *LecRK6.1*, *WRKY20*, and *Cupi4* compared to control cucumbers. These results suggest that the defense response of BABA in cucumbers is associated with SA signaling pathway-dependent systemic acquisition resistance (SAR) involved in plant resistance mechanisms. Ahn et al. [12] conducted a molecular assessment of the effects of FLC homologs and coordination regulators on the flowering response of the cabbage (*Brassica oleracea* var. *capitata*) genotype. The researchers investigated *BoGI*, *BoCOLAIR*, and *BoVIN3*, which are inhibitors of early (CAB1)-, middle (CAB3)-, and late (CAB5)-flowering cabbage genotypes, to clarify the flowering mechanisms in cabbage. An analysis of allele-specific amplified genomic DNA and various sequence alignments demonstrated that maximum insertion and deletion, particularly in CAB3 and CAB5, influenced cabbage flowering behavior. Phylogenetic studies have shown that *BoFLC1*, *2*, and *3* of the CAB1, 3, and 5 genotypes showed the highest homology to other Brassica species, while CAB3 and 5 were the most similar. Expression investigations revealed that essential CAB5 downregulated all *BoFLC* genes compared to CAB3, in contrast to CAB3. These results suggest that structural changes in the three genotypes, such as BoFLC2 and COOLAIR, may affect cabbage flowering time. Xiong et al. [13] reported that knockout of the chlorophyll oxygenase gene *OsCAo1* reduces cold resistance in rice seedlings. In the rice genome, two genes (*OsCAo1* and *OsCAo2*) are highly homologous to *AtCAo* [13]. The *OsCAo1* knockout mutant line exhibited light green leaves, indicating Chlb deficiency, but the knockout mutation in *OsCAo2* did not alter leaf color [14]. The presence of cis-acting elements involved in cold reactivity (LTR) in the *OsCAo1* promoter suggests that *OsCAo1* is probably a cold-responsive gene. The researchers stated that the gene expression level of *OsCAo1* is usually inhibited by low temperatures during the day and promoted by low temperatures at night. In rice, the *OsCAo1* knockout mutant line, produced by CRISPR-Cas9 technology, exhibited significantly weakened chilling resistance at the seedling stage. *OsCAo1* dysfunction led to the accumulation of reactive oxygen species and malondialdehyde, increased relative electrolyte leakage, and decreased antioxidant gene expression under chilling stress. In addition, functional deficiency of *OsCAo1* caused more severe damage to chloroplast morphologies, such as abnormal grana thylakoid stacking due to low temperatures. In addition, rice yields were reduced in *OsCAo1* knockout mutant lines. Thus, a high expression of *OsCAo1* likely increases both rice yield and chill tolerance, providing a strategy for growing chill-resistant rice varieties with high yields. In fact, it seems that academic and industrial research on molecular breeding technology take different paths depending on the area of interest. Unfortunately, we need to build a database of varieties and products obtained from various breeding technologies so that studies on molecular breeding technologies carried out in academia and industry can be linked to each other. We believe that continuous collaborative efforts between academia and industry are necessary to solve this problem.
